# Stimulus discrimination and association with Hebbian cell assemblies

**DOI:** 10.1186/1471-2202-16-S1-P287

**Published:** 2015-12-18

**Authors:** Johannes M Auth, Timo Nachstedt, Christian Tetzlaff, Florentin Wörgötter

**Affiliations:** 1 Third Institute of Physics, Georg-August-University, Göttingen, 37077, Germany; 2 Bernstein Center for Computational Neuroscience, Göttingen, 37077, Germany

## 

Learning or the formation of memories is a key component of biological intelligence. One distinguishes between different types of memory as, for instance, the declarative memory denoting the storage of facts and concepts which are extracted from perceived stimuli. Thereby, an important question is how the brain distinguishes between stimuli that should be associated and those to be discriminated. Investigating this mechanism, in psychophysical experiments, rats were exposed to gradually morphed environments while recording activity in hippocampal place cells [1,2]. Stability of the recorded place-dependent activation patterns was dependent on the order in which the different environments were explored. In other words, stimulus discrimination or association depends on the order of presentation. Theoretical models of memory rely on the hypothesis that memorization is implemented through activity-dependent synaptic plasticity. Environmental patterns are reflected by correlated activity in subgroups of neurons leading to high recurrent synaptic efficacies among them. These groups of highly interconnected neurons are named cell assemblies (CAs). This long-standing concept with its basic ability of pattern completion is a promising approach to explain several cognitive phenomena [3]. Combining the theory of CAs with the principle of self-organizing maps (SOMs) [4], we present a biologically plausible network model able to reproduce the upper experimental findings.

Basically our model consists of three populations of neurons. An input population projects through random excitatory connections on a recurrent layer. Neurons within the layer interact by excitatory synapses. Competition is introduced by an inhibitory population mutually interconnected with every layer neuron. For all neurons, we use a rate-coded neuron model with sigmoidal transfer functions. The plasticity model combines (Hebbian) synaptic plasticity and synaptic scaling (SPASS) [5]. Stimuli are inserted as activity patterns in the input population passing them on via afferent plastic synapses. Recurrent lateral connections in the layer reach a circular neighborhood of fixed range and are also plastic. The incoming excitatory synapses the inhibitory population receives as well as its outgoing connections are non-plastic. In agreement with SOM, our concept maps sufficiently differing stimuli to different representations (CAs) in the layer.

Comparable to the experimental procedures described above, we define two non-overlapping activity patterns A and B. According with [1], first we transform stimulus A gradually into B with a short pause between each step of the transition sequence. After A lead to the formation of one memory representation, or rather a single CA, this CA is maintained throughout the following learning protocol. Secondly, the protocol of [2] demands a random presentation of the transition patterns. In this case, we observe the formation of multiple representations. Thus, our model reproduces the experimental findings described above. In contrast to existing theoretical studies [6], it does not require stimulus-dependent modifications of the plasticity rule itself. It further allows for investigating the influence of different variations in the learning protocol as, for instance, of the stimulus presentation time.
